# Construction of a SUMOylation regulator‐based prognostic model in low‐grade glioma

**DOI:** 10.1111/jcmm.16553

**Published:** 2021-05-05

**Authors:** Xiaozhi Li, Yutong Meng

**Affiliations:** ^1^ Department of Neurosurgery Shengjing Hospital of China Medical University Shenyang China; ^2^ Department of Stomatology Shengjing Hospital of China Medical University Shenyang China

**Keywords:** low‐grade glioma, post‐translational modification, prognosis, SUMOylation

## Abstract

Low‐grade glioma (LGG) is an intracranial malignant tumour that mainly originates from astrocytes and oligodendrocytes. SUMOylation is one of the post‐translational modifications but studies of SUMOylation in LGG is quite limited. Transcriptome data, single nucleotide variant (SNV) data and clinical data of LGG were derived from public databases. The differences between the expression of SUMOylation regulators in LGG and normal brain tissue were analysed. Cox regression was used to construct a prognostic model in the training cohort. Kaplan‐Meier survival curves and ROC curves were plotted in the training and the validation cohort to evaluate the effectiveness of the prognostic model. GO and KEGG analyses were applied to preliminarily analyse the biological functions. Compared with normal brain tissue, SENP1 and SENP7 were up‐regulated and SENP5 was down‐regulated in LGG. SUMOylation regulators may be involved in functions such as mRNA splicing, DNA replication, ATPase activity and spliceosome. One prognostic model was established based on the 4 SUMOylation regulator‐related signatures (RFWD3, MPHOSPH9, WRN and NUP155), which had a good predictive ability for overall survival. This study is expected to provide targets for the diagnosis and treatment of low‐grade glioma.

## 1 INTRODUCTION

Low‐grade glioma (LGG) is an intracranial malignant tumour that mainly originates from astrocytes and oligodendrocytes. Compared with glioblastoma, it often affects the health of patients in a chronic and long‐term manner.^1^ Among them, IDH mutation and 1p/19q co‐deletion have been found very early in LGG and have been widely used in molecular classification,^2^, ^3^ but this is far from being able to explain the specific molecular mechanism of LGG. Surgery, radiotherapy and chemotherapy are the main treatments for LGG.^4^, ^5^, ^6^ How to eliminate tumour cells as much as possible and maximize the quality of life of patients remains a major challenge.

SUMOylation is one of the post‐translational modifications of proteins independent of phosphorylation, glycosylation and ubiquitylation.^7^, ^8^ The three‐dimensional structure of SUMO is similar to ubiquitin, but the amino acid sequence is quite different. The SUMOylation process includes the catalysis of three kinds of enzymes, namely SUMO activating enzyme (E1, including SAE1, UBA2), SUMO conjugating enzyme (E2, including UBE2I) and SUMO ligase (E3, including PIAS1, PIAS2, PIAS3, PIAS4, RANBP2). In addition, deSUMOylation is the opposite effect of SUMOylation and is catalysed by SUMO proteases (SENP1, SENP2, SENP3, SENP5, SENP6, SENP7, USPL1). It has been reported that UBE2I expression is up‐regulated in glioblastoma, accompanied by activation of SUMOylation.^9^ However, studies of SUMOylation in LGG were quite limited.

In this study, the expression of SUMOylation regulators in LGG was analysed by high‐throughput sequencing database, and the related molecules of SUMOylation regulators were screened to construct a prognostic model for LGG. This work aimed to provide targets for the study of the pathogenesis of low‐grade glioma.

## 2 MATERIALS AND METHODS

### 2.1 Acquisition of data

The single nucleotide variant (SNV) data of LGG was derived from the TCGA database (https://cancergenome.nih.gov/). One of transcriptome data of LGG was derived from the TCGA database and the corresponding clinical information of TCGA‐LGG was acquired from cBioportal database (https://www.cbioportal.org/). The other independent transcriptome data and clinical data of LGG were acquired from CGGA database (http://www.cgga.org.cn/, including mRNAseq_325 and mRNAseq_693). ‘sva’ package of R language was applied to integrate two CGGA data sets to eliminate batch effect. The intersection genes of TCGA and CGGA data sets were used for subsequent analysis. Samples with incomplete survival data were excluded from survival analysis.

### 2.2 The expression of SUMOylation regulators in LGG

The R language ‘maftools’ package was conducted to detect SNV information. The expression of SUMOylation regulators in LGG was extracted from the TCGA data set. The gene expression differences between LGG and normal brain tissue were analysed by Wilcoxon rank‐sum test using | log_2_ FC | > 0.5 and *P* <.05 as threshold, and a heatmap of SUMOylation regulators was drawn accordingly. The expressions of SUMOylation regulators in IDH wild‐type and IDH mutant‐type subgroups were further analysed.

### 2.3 Construction and verification of a prognostic model based on SUMOylation regulator‐related signatures

In this study, the TCGA‐LGG was used as the training group and the CGGA‐LGG was used as the validation group for the construction and verification of the risk model. For the CGGA data sets, the same formula was inherited from the TCGA dataset during the validation of the risk model.

First, the correlations between differentially expressed genes and differentially expressed SUMOylation regulators were calculated by Pearson's method in the training cohort. SUMOylation regulator‐related signatures were determined by Pearson's correlation when coefficient was greater than 0.8 and *P* <.05. Next, univariate Cox regression was applied to select prognosis‐related genes. The prognostic model was built by stepwise multivariate Cox regression. Signatures with lowest Akaike Information Criterion (AIC) value were included to avoid over‐fitting. Finally, Kaplan‐Meier survival curves and ROC curves were plotted in the two independent data sets (TCGA‐LGG and CGGA‐LGG) to evaluate the effectiveness of the prognostic model. For short, the high‐risk and low‐risk groups were separated by median value of risk scores. ‘survival’ package of R language was used to compare the overall survival difference. Moreover, ROC curves were plotted by ‘timeROC’ package of R language.

### 2.4 Functional enrichment analysis

To preliminarily analyse the biological functions of the molecular involvement of SUMOylation regulators, GO and KEGG enrichment analyses were performed using the R language ‘clusterProfiler’ package.

### 2.5 Statistical analysis

All statistics and graphics were implemented based on R language (4.0.2). When *P* <.05, it is considered statistically different.

## 3 RESULTS

### 3.1 SNV of the SUMOylation regulators

**FIGURE 1:**
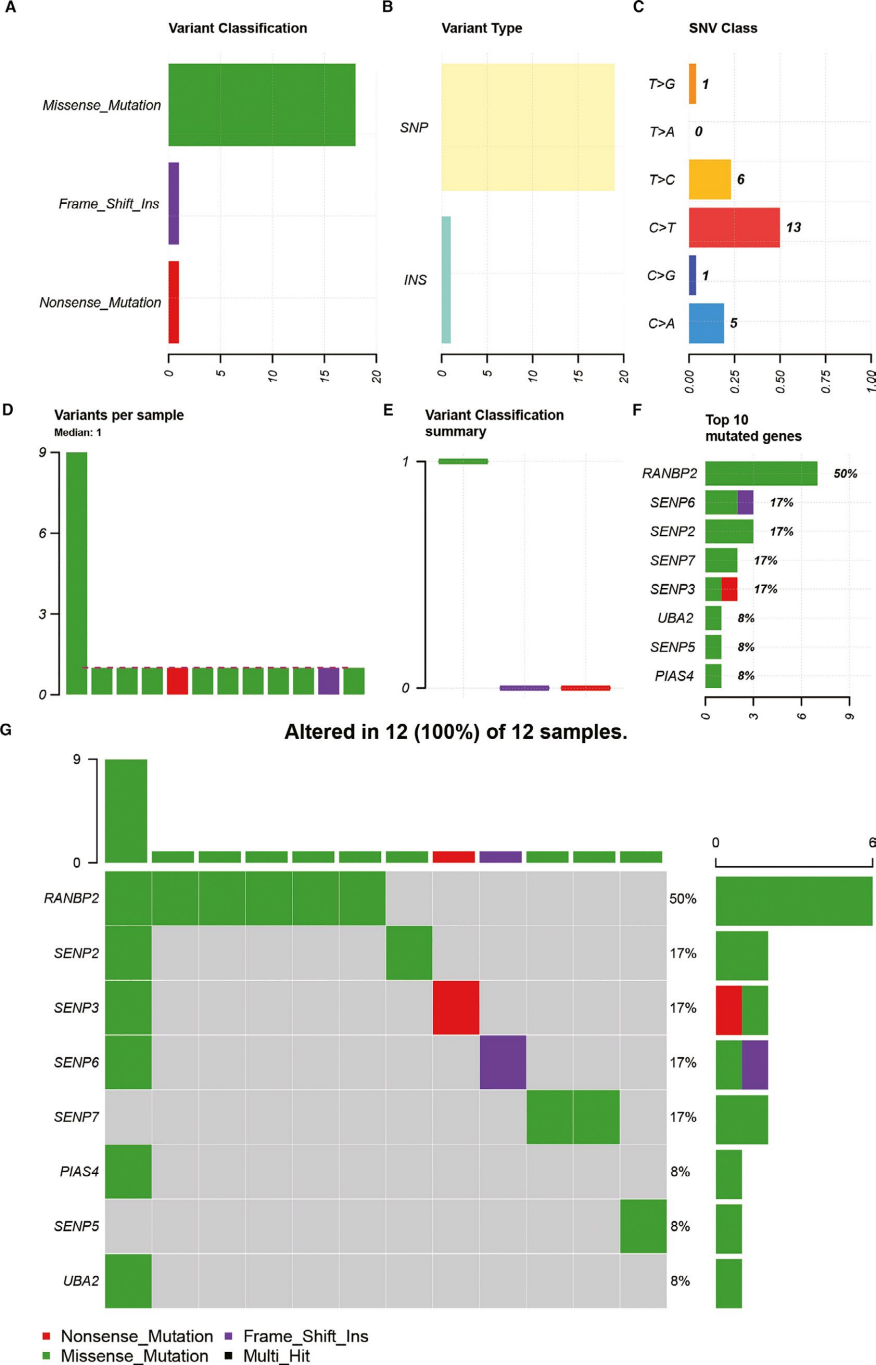
Overview of SUMOylation regulator mutation profiles in LGG. A‐C, Missense mutation, SNP and C > T were more common in LGG mutation. D, Overall distribution of mutations in each sample. E, Missense mutation existed in all 12 mutation samples. F, Mutation of RANBP2 was the most frequent. G, Distribution relationship between mutant genes and LGG samples

In this study, mutations of 15 SUMOylation regulators in LGG were detected in the TCGA database. A total of 12 (2.372%) of 506 LGG samples showed mutations in SUMOylation regulators (Figure 1). Missense mutation, SNP and C > T were the most common forms of mutation (Figure 1A‐C). Figure 1D showed the overall distribution of mutations in each sample. Missense mutation existed in all 12 mutation samples (Figure 1E). Among the 15 SUMOylation regulators, RANBP2, SENP6, SENP2, SENP7, SENP3, UBA2, SENP5 and PIAS4 showed mutations in SNV. Besides, mutation of RANBP2 was the most frequent (Figure 1F). Figure 1G showed the distribution relationship between mutant genes and LGG samples in the form of a waterfall plot.

### 3.2 Differentially expressed SUMOylation regulators

**FIGURE 2:**
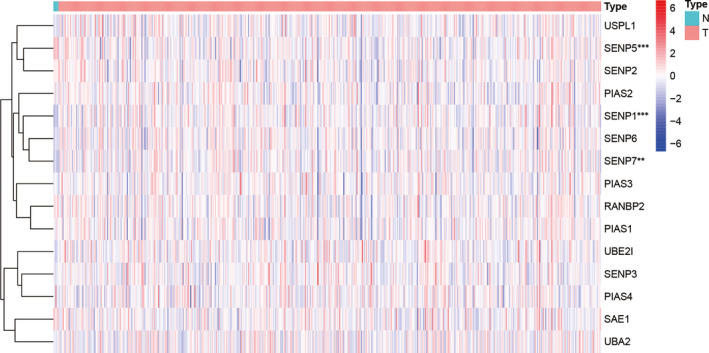
Heatmap of SUMOylation regulators between LGG and normal brain tissue

**FIGURE 3:**
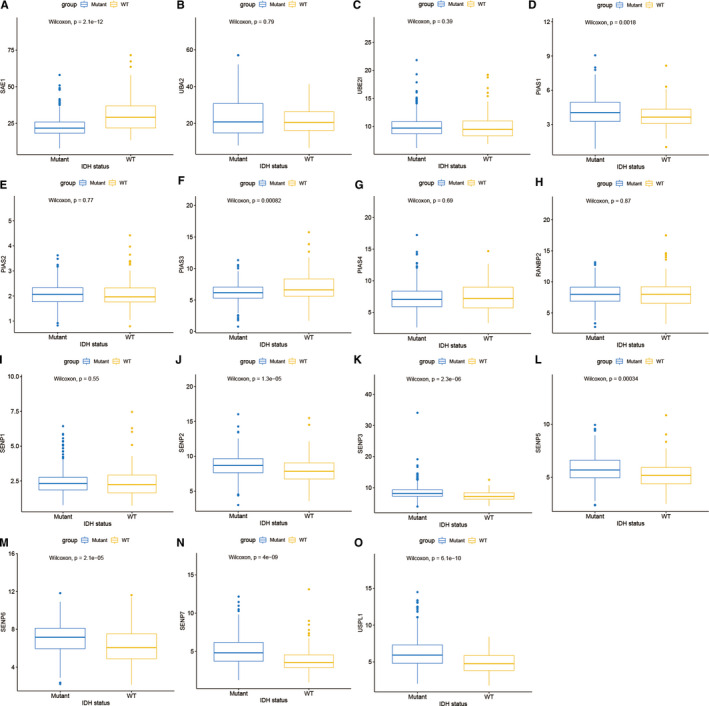
Expression of SUMOylation regulators in different IDH status

The clinical characteristics of LGG samples with complete survival data were shown in the Table S1. Compared with normal brain tissue, SENP1 and SENP7 were up‐regulated and SENP5 was down‐regulated in LGG (| log_2_ FC | > 0.5 and *P* <.05), as shown in Figure S1. The heatmap of SUMOylation regulator expressions in LGG was shown in Figure 2. In addition, SUMOylation regulator expression distributions in IDH wild‐type and IDH mutant‐type subgroups were statistically analysed in this study, and the results were shown in Figure 3. Compared with IDH wild‐type subgroup, the expressions of SAE1, PIAS3 and USPL1 increased in IDH mutant‐type subgroup, while the expressions of PIAS1, SENP2, SENP3, SENP5, SENP6 and SENP7 decreased.

### 3.3 Functional enrichment analysis

**FIGURE 4:**
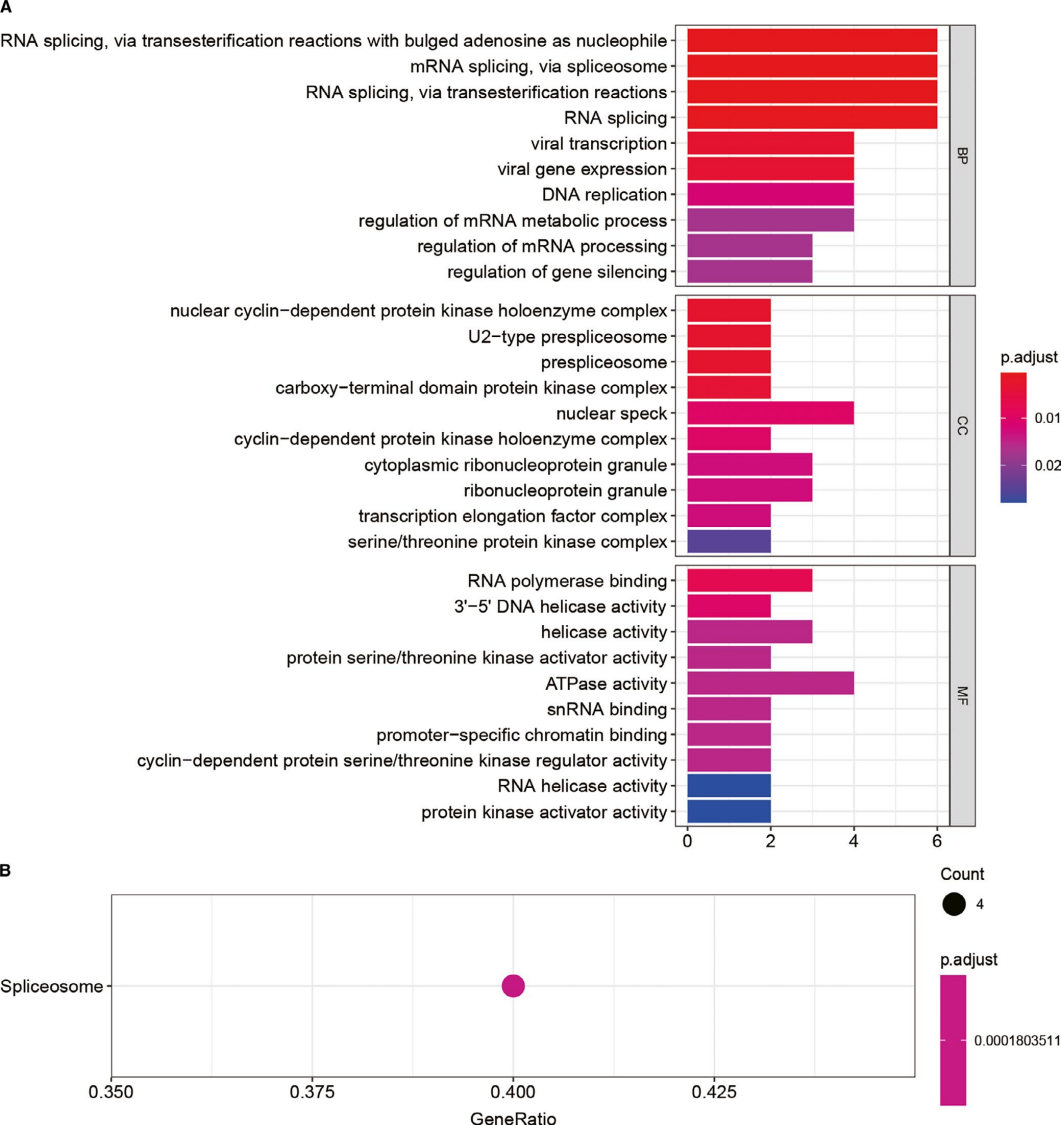
Functional enrichment results of SUMOylation regulators. A, GO enrichment results showed that SUMOylation regulators were enriched in items such as mRNA splicing, DNA replication, ATPase activity. B, KEGG enrichment results showed that SUMOylation regulators were enriched in spliceosome

SUMOylation regulator‐related signatures were screened by Pearson's correlation coefficient >0.8 and *P* <.05 and their biological functions were explored by GO and KEGG enrichment methods. The result was shown in Figure 4. SUMOylation regulators may be involved in functions such as mRNA splicing, DNA replication, ATPase activity and spliceosome.

### 3.4 Construction and verification of a prognostic model based on SUMOylation regulator‐related signatures

**FIGURE 5:**
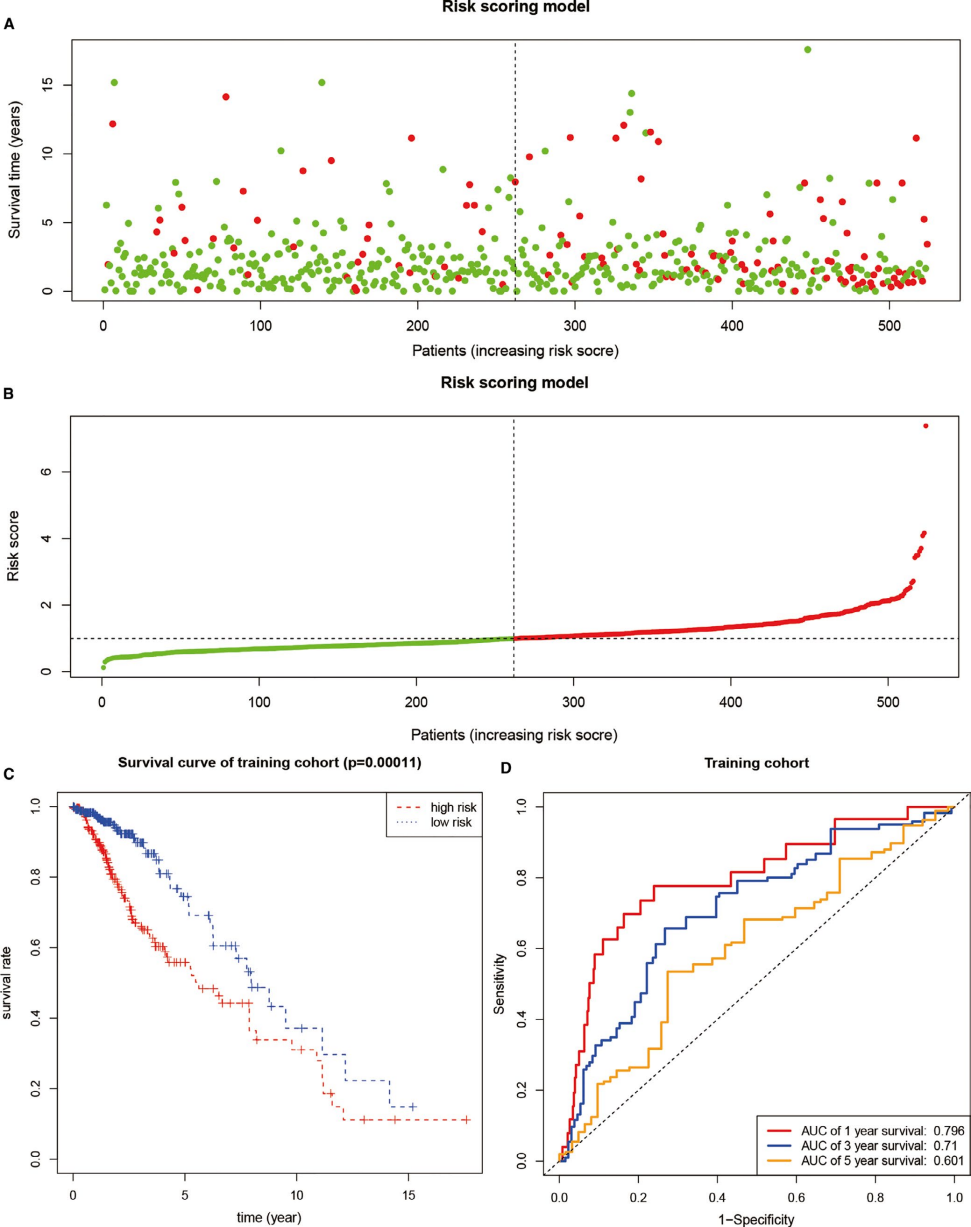
Prognostic model based on SUMOylation regulator‐related signatures. A and B, The overview and overall survival and risk scores. C, Kaplan‐Meier survival curve of the training group. D, ROC curve of the training group

**FIGURE 6:**
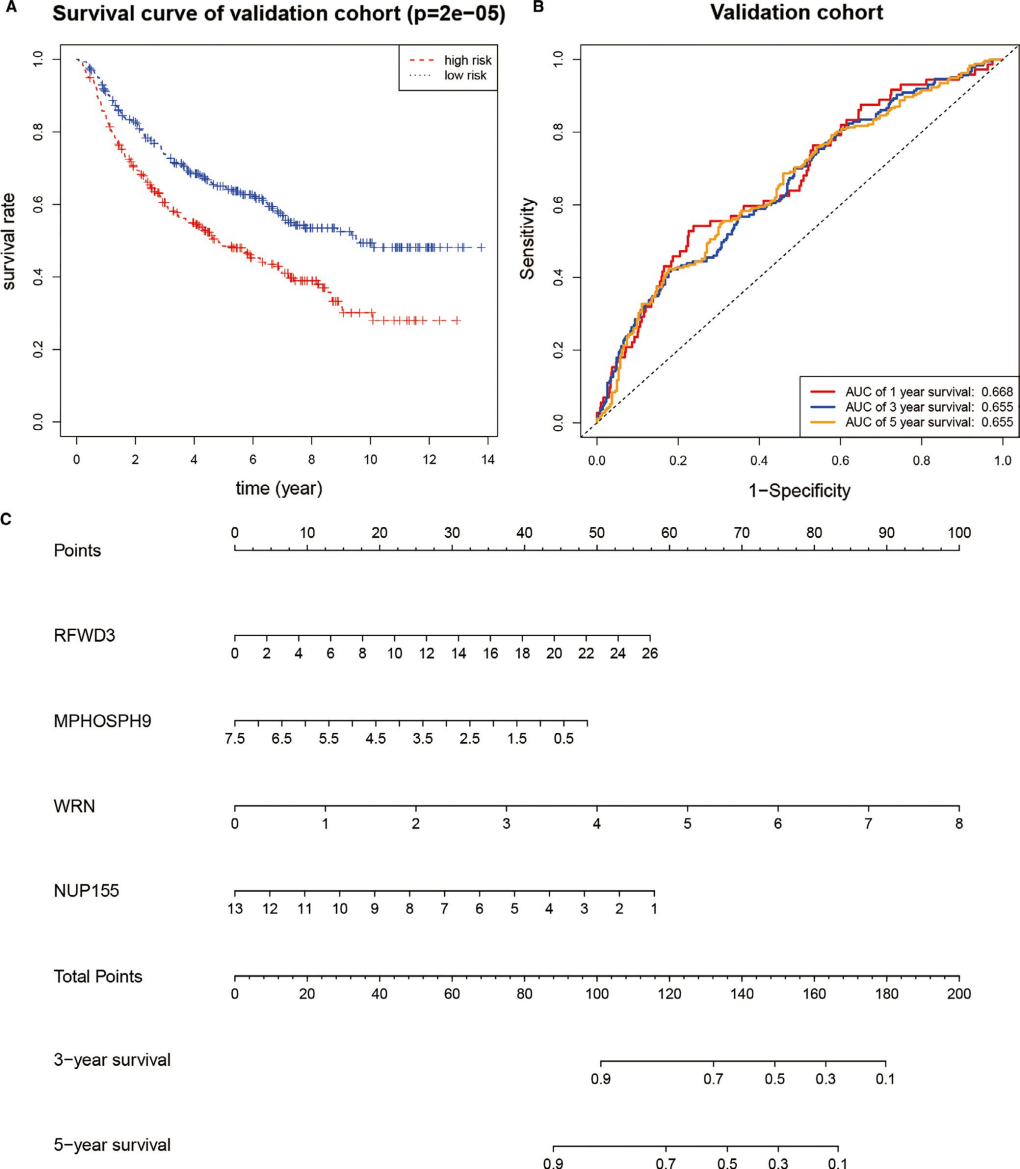
Validation of the prognostic model. A, Kaplan‐Meier survival curve of the validation group. B, ROC curve of the validation group. C, Nomogram of the prognostic model based on 4 SUMOylation regulator‐related signatures

Among the SUMOylation regulator‐related signatures, a total of 14 differentially expressed genes in LGG were further extracted, namely DHX9, RFWD3, DDX46, SF3B1, TIA1, TMPO, SUZ12, MPHOSPH9, WRN, CTDSPL2, PRPF40A, ARID2, ATAD2B and NUP155. Then, a prognostic model was built by Cox regression. The risk score could be expressed as Risk score =0.947 * Expression _RFWD3_ ‐ 0.655 * Expression _MPHOSPH9_ + 1.204 * Expression _WRN_ – 1.135 * Expression _NUP155_. The risk score and survival status of all samples were shown in Figure 5A‐B. The Kaplan‐Meier survival curve of the training group suggested that patients with high‐risk scores suffered worse survival status than patients with low‐risk scores (*P* <.001, Figure 5C). The ROC curve of the training group showed that the 1‐year AUC was 0.796, the 3‐year AUC was 0.710, and the 5‐year AUC was 0.601. In the validation group, patients with high‐risk scores also had worse survival status than patients with low‐risk scores (*P* <.001, Figure 6A). The ROC curve of the training group indicated that the 1‐year AUC was 0.688, the 3‐year AUC was 0.655, and the 5‐year AUC was 0.655. Moreover, a nomogram was drawn based on the prognostic model, as shown in Figure 6C.

### 3.5 Independent prognostic value of the risk score

Next, we conducted univariate and multivariate Cox analysis to evaluate the independent prognostic value of risk score based on the prognostic model. The results showed that the risk score was an independent prognostic factor apart from age and radiotherapy of LGG, as shown in Figure S2.

## 4 DISCUSSION

The treatment of LGG is a comprehensive treatment based on maximum surgical resection, but progression of deterioration is still inevitable, and patients often have adverse outcomes such as multicentre recurrence and meningeal dissemination.^10^, ^11^, ^12^ SUMOylation, as a covalent modification, occurs mainly in lysine residues^13^ and plays an important role in maintaining protein stability and stress response.^14^ The dynamic regulation of SUMOylation and deSUMOylation is also involved in tumour molecular regulation.^15^, ^16^, ^17^ For example, the SUMOylation of P53 at K386 can prevent it from being acetylated by p300, but P53 acetylated by p300 can still be SUMOylated and reduce the DNA binding inhibited by SUMOylation.^18^ In esophageal squamous cell carcinoma, HSP27 can directly interact with SUMO2/3, and SUMOylation of HSP27 enhances tumour cell proliferation, migration and invasion.^19^ However, the research on the regulatory mechanism of SUMOylation in glioma, especially LGG is quite limited.

This study first evaluated the distribution of SNV of the SUMOylation regulators in LGG. The percentage of all samples with SUMOylation regulator mutation was 2.372%. Furthermore, we analysed the differential expression of SUMOylation regulators between LGG and normal brain tissue. It was found that SENP1 and SENP7 were up‐regulated, and SENP5 was down‐regulated. In order to explore the possible involvement of SUMOylation regulators in biological functions, we used functional enrichment analysis to analyse the SUMOylation regulator‐related signatures and found that SUMOylation regulators might participate in biological functions such as mRNA splicing, DNA replication, ATPase activity and spliceosome. DHX9, RFWD3, DDX46, SF3B1, TIA1, TMPO, SUZ12, MPHOSPH9, WRN, CTDSPL2, PRPF40A, ARID2, ATAD2B and NUP155 were differentially expressed and correlated with SUMOylation regulators. A prognosis model was constructed based on RFWD3, MPHOSPH9, WRN and NUP155. The Kaplan‐Meier survival curves and ROC curves of the training group and the validation group both showed the prognostic model had a good predictive ability for overall survival of LGG.

SENP1, SENP7 and SENP5 were SUMOylation regulators we identified which differentially expressed in LGG. It has been reported that SENP1 is positively correlated with the malignant degree of glioma. Down‐regulated SENP1 can inhibit the phosphorylation of IκBα and Akt and inhibit the expression of downstream BCL XL and CyclinD1.^20^ However, there are few reports on the relationship between SENP5/SENP7 and LGG. In addition, the SUMOylation regulator‐related signatures we identified have been reported in some studies. For instance, SUZ12, one of the components of the PRC2 complex, can directly bind with conjugating enzyme UBE2I and be SUMOylated.^21^ Furthermore, DHX9 is a member of the DEAH helicase family and it can interact with UBE2I to bind to SUMO1.^22^ However, the molecular mechanism of these molecules in LGG is rarely studied and remains to be explored.

This study still has certain limitations. For example, more basic experiments can better prove the conclusion and explore the specific molecular mechanisms of these molecules involved in SUMOylation.

## 5 CONCLUSIONS

This study demonstrated the expression of SUMOylation regulators in LGG. SUMOylation regulators might be involved in biological functions such as mRNA splicing, DNA replication, ATPase activity, spliceosome. One prognostic model was established based on the 4 SUMOylation regulator‐related signatures (RFWD3, MPHOSPH9, WRN and NUP155), which had a good predictive ability for overall survival. This study is expected to provide targets for the diagnosis and treatment of low‐grade glioma.

## CONFLICT OF INTEREST

Not applicable.

## AUTHOR CONTRIBUTIONS

**Xiaozhi Li:** Conceptualization (equal); Data curation (equal); Formal analysis (equal); Funding acquisition (lead); Investigation (equal); Methodology (equal); Project administration (equal); Resources (equal); Software (equal); Supervision (equal); Validation (equal); Visualization (equal); Writing‐original draft (equal); Writing‐review & editing (equal). **Yutong Meng:** Conceptualization (equal); Data curation (equal); Formal analysis (equal); Funding acquisition (supporting); Investigation (equal); Methodology (equal); Project administration (equal); Resources (equal); Software (equal); Supervision (equal); Validation (equal); Visualization (equal); Writing‐original draft (equal); Writing‐review & editing (equal).

## ETHICAL APPROVAL

The data came from public databases and no more ethical approval was needed.

## Supporting information

Figure S1

Figure S2

Table S1

## Data Availability

Data were acquired from TCGA database (https://cancergenome.nih.gov/), CGGA database (http://www.cgga.org.cn/) and cBioportal database (https://www.cbioportal.org/).
