# Supplementary figures and images for: Construction of a SUMOylation regulator‐based prognostic model in low‐grade glioma
Source: J Cell Mol Med. 2021 May 5;25(12):5434–42. doi: 10.1111/jcmm.16553 (PMC8184686; doi:10.1111/jcmm.16553)

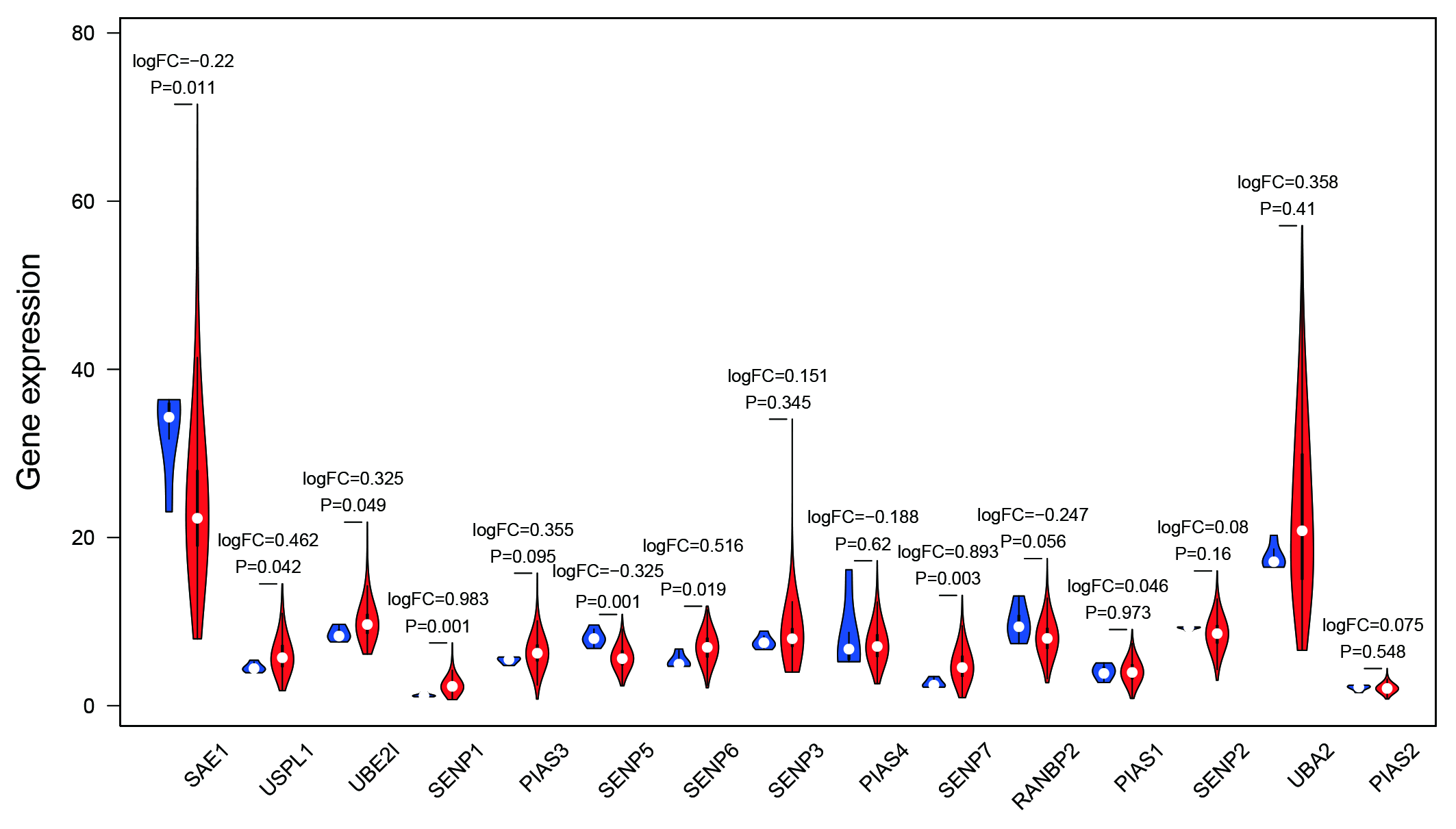

Supplement: Supplementary file 1 — Figure S1 [file JCMM-25-5434-s003.tif]

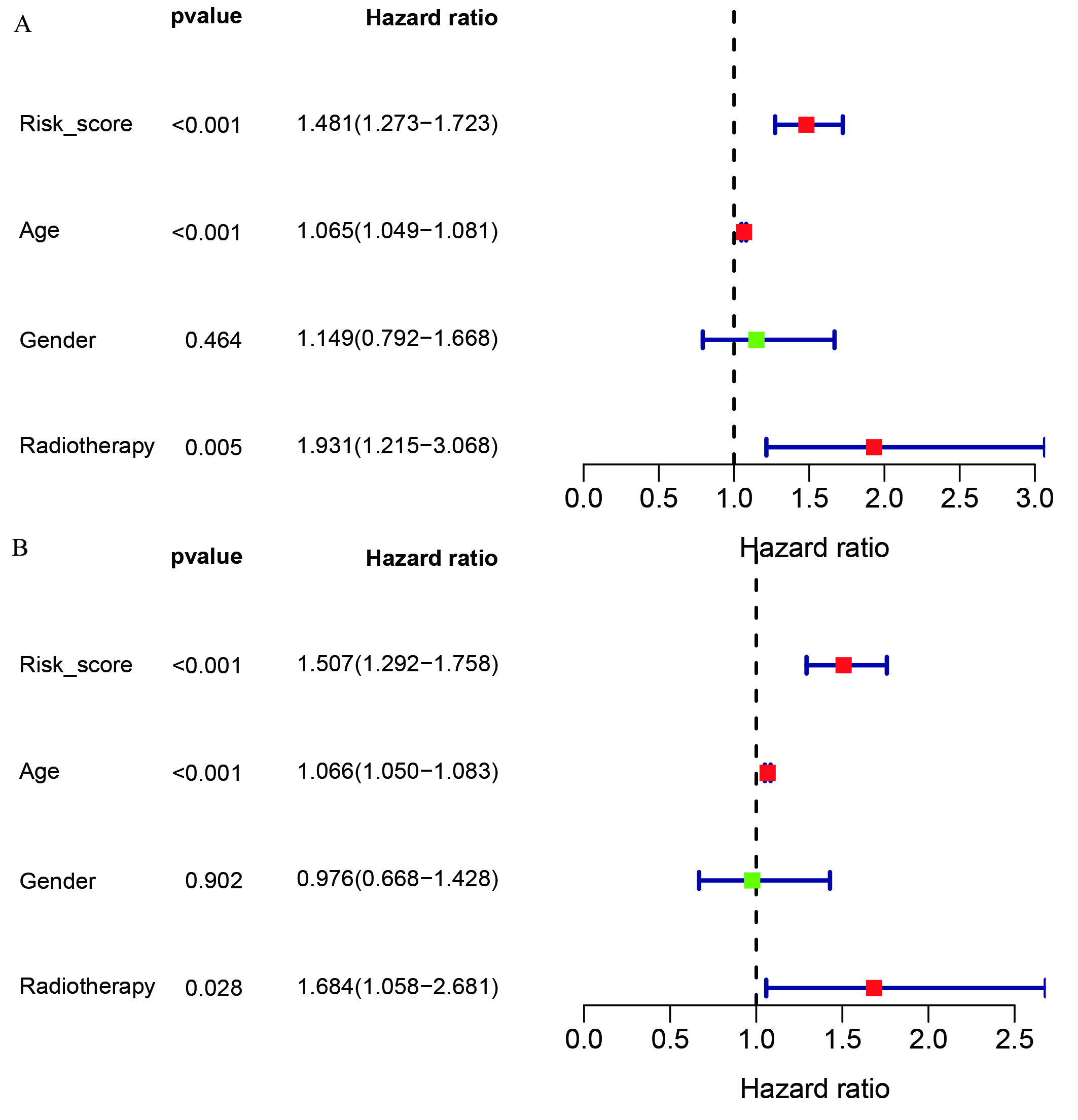

Supplement: Supplementary file 2 — Figure S2 [file JCMM-25-5434-s001.tif]
