# Supplementary material for: Construction of a SUMOylation regulator‐based prognostic model in low‐grade glioma
Source: J Cell Mol Med. 2021 May 5;25(12):5434–42. doi: 10.1111/jcmm.16553 (PMC8184686; doi:10.1111/jcmm.16553)
Supplement: Supplementary file 3 — Table S1 [file JCMM-25-5434-s002.doc]

| Table S1. Clinical characteristics of datasets | | | |
| --- | --- | --- | --- |
|  | TCGA | CGGA- mRNAseq_325 | CGGA- mRNAseq_693 |
| Number of cases | 506 | 172 | 420 |
| Age (years) | 42.99±13.40 | 40.45±10.84 | 40.32±10.36 |
| Gender |  |  |  |
| Male | 280 | 106 | 235 |
| Female | 226 | 66 | 185 |
| Vital status |  |  |  |
| Alive | 397 | 82 | 223 |
| Dead | 109 | 90 | 197 |
| Radiation |  |  |  |
| Yes | 292 | 139 | 308 |
| No | 179 | 29 | 99 |
| Unclear | 35 | 4 | 13 |
